# Stridor and Dysphagia Unmasking an Aberrant Right Subclavian Artery in a Toddler

**DOI:** 10.5811/cpcem.61391

**Published:** 2026-07-20

**Authors:** Masato Yasuda, Tomoya Ito

**Affiliations:** Aichi Children’s Health and Medical Center, Division of Pediatric Emergency Medicine, Morioka-cho, Obu City, Aichi, Japan

**Keywords:** aberrant right subclavian artery, dysphagia, pediatric emergency, stridor, vascular ring

## Abstract

**Case Presentation:**

A one-year-old girl presented with acute-onset dyspnea persistent for two days and recurrent choking episodes. Physical examination revealed stridor and sub-costal retractions. Despite initial treatment with inhaled nebulized epinephrine, stridor persisted. Lateral chest radiography finding indicated tracheal stenosis. Contrast-enhanced neck and chest computed tomography (CT) showed an aberrant right subclavian artery compressing the esophagus posteriorly, causing esophageal stenosis with associated food residue accumulation. The proximally dilated portion of the esophagus caused mass effect with compression of the trachea, resulting in tracheal stenosis. Despite medical management, the patient experienced recurrent stridor and could only swallow liquids. Therefore, surgical translocation of the right subclavian artery to the right common carotid artery was performed. Postoperatively, the patient swallowed age-appropriate food, and the stridor resolved.

**Discussion:**

The most common cause of stridor in children is viral croup, which is typically treated with nebulized epinephrine and corticosteroids in moderate to severe cases. However, recurrent or persistent stridor with dysphagia should raise concern for vascular anomalies (eg, aberrant right subclavian artery), bacterial tracheitis, epiglottitis, retropharyngeal abscess, foreign body aspiration, mediastinal tumors, or other compressive pathologies. Early symptomatic presentation with dysphagia and stridor in young children is unusual, as aberrant right subclavian artery symptoms typically develop after age 40. When clinical presentation deviates from typical croup, particularly if associated with dysphagia, ultrasound, magnetic resonance imaging, or contrast-enhanced CT can identify vascular anomalies. Surgical intervention is indicated for symptomatic aberrant right subclavian artery when symptoms persist despite medical management (nutrition monitoring, feeding support, airway support, and growth assessment).

## CASE PRESENTATION

**Image 1:**
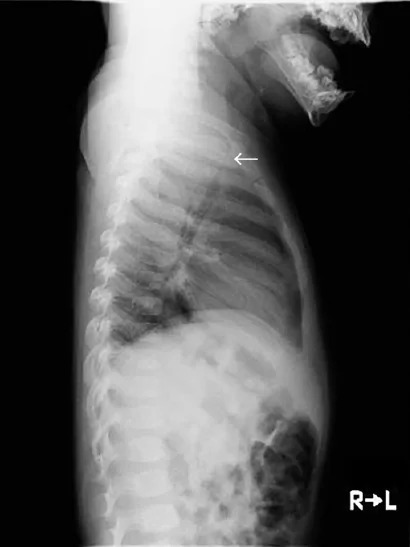
Lateral plain chest radiograph showing tracheal stenosis in a toddler who presented with acute-onset dyspnea (arrow).

A one-year-old girl presented to the emergency department with acute-onset dyspnea persistent for two days, which developed after eating. Over the preceding two weeks, she had experienced recurrent episodes of choking while eating, accompanied by stridor and respiratory distress. Physical examination revealed prominent stridor and subcostal retractions that worsened during crying, with oxygen saturation maintained at 98% on room air and a respiratory rate of 24 breaths per minute. Following nebulized epinephrine inhalation, the symptoms temporarily improved, but the stridor persisted. Lateral plain chest radiography revealed tracheal stenosis (Image 1).

**Image 2:**
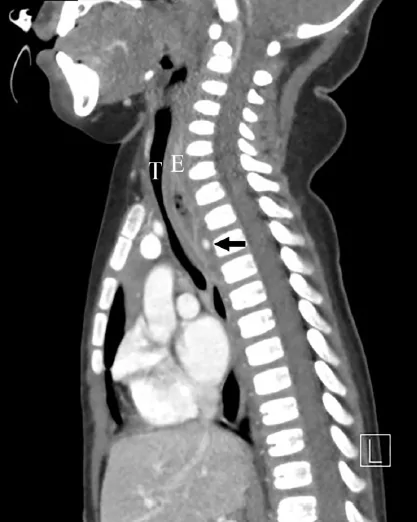
Sagittal contrast-enhanced computed tomography image shows tracheal stenosis (T) due to a dilated esophagus (E) that had expanded because of food residue accumulation above the esophagus segment, which is compressed by an aberrant right subclavian artery (arrow).

**Image 3:**
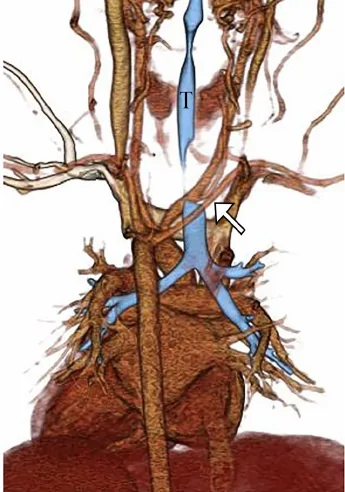
Three-dimensional computed tomography image showing tracheal stenosis (T) and an aberrant right subclavian artery traversing dorsally (arrow).

Contrast-enhanced neck and chest computed tomography (CT) was performed, revealing an aberrant right subclavian artery compressing the esophagus posteriorly, causing esophageal stenosis and accumulation of food residues above the stenotic segment of the esophagus (Image 2 and 3).

The patient was treated with nebulized epinephrine and intravenous dexamethasone; however, she could only tolerate liquids and continued to experience recurrent stridor. Therefore, surgery was performed to translocate the right subclavian artery to the right common carotid artery. Postoperatively, she was able to swallow age-appropriate food, and the stridor resolved.

*CPC-EM Capsule*

What do we already know about this clinical entity?

*Aberrant right subclavian artery is the most frequent congenital anomaly of the aortic arch.*

What is the major impact of the image(s)?

*Contrast-enhanced computed tomography shows a retroesophageal aberrant right subclavian artery causing esophageal stenosis.*

How might this improve emergency medicine practice?

*Persistent or recurrent stridor with dysphagia in children should prompt consideration of vascular anomalies and use of appropriate imaging.*

## DISCUSSION

Aberrant right subclavian artery is the most frequent congenital anomaly of the aortic arch, with a prevalence of approximately 1% in the general population.^1^ Although only approximately 5% of patients become symptomatic, an aberrant right subclavian artery can compress adjacent structures, such as the esophagus and trachea, resulting in dysphagia lusoria (34%), dyspnea (25%), chest pain (16%), cough (8%), and upper limb claudication (5%).^1^,^2^

In our case, the aberrant right subclavian artery followed a retroesophageal course, which is the most common, directly compressing the esophagus followed by the trachea, explaining the patient’s combined dysphagia and stridor.^1^ Early symptomatic presentation with both dyspnea and dysphagia is unusual in young children.^2^ Symptoms typically develop after age 40 when aortic wall rigidity increases because of atherosclerosis.^1^

Croup is the most common cause of pediatric stridor. It is typically treated with epinephrine inhalation and, in moderate to severe cases, with systemic corticosteroid administration.^3^ However, recurrent or persistent stridor with dysphagia should raise concern for vascular anomalies (eg, aberrant right subclavian artery), bacterial tracheitis, epiglottitis, retropharyngeal abscess, foreign body aspiration, mediastinal tumors, or other compressive pathologies.^3^,^4^

When the clinical presentation deviates from typical croup manifestations, particularly with dysphagia, physicians should consider plain chest radiography, including posterior-anterior and lateral views, followed by ultrasonography, magnetic resonance imaging, or contrast-enhanced CT.^2^ Prior to imaging, airway patency and adequate ventilation must be assessed. Contrast-enhanced CT is essential to identify vascular anomalies and guide management planning.

For symptomatic aberrant right subclavian artery, initial medical management includes nutrition monitoring, feeding support (eg, nasogastric tube), airway support, and growth assessment.^2^ Surgical treatment, particularly subclavian artery translocation by pediatric cardiovascular surgeons, is indicated for symptomatic aberrant right subclavian artery when dysphagia with progressive weight loss or significant stridor persist despite medical management.^2^,^5^ Untreated cases are associated with recurrent pneumonia, obstructive emphysema, and aortic dissection in adulthood.^1^
